# Physiological and biochemical responses of hybrid maize (*Zea mays L*.) varieties grown under heat stress conditions

**DOI:** 10.7717/peerj.14141

**Published:** 2022-09-21

**Authors:** Timucin Tas

**Affiliations:** Kepsut Vocational High School, Balikesir University, Balikesir, Turkey

**Keywords:** Maize, Heat stress, Cell membrane damage, Chlorophyll

## Abstract

Maize (*Zea mays L*.) is the second most commonly produced and consumed crop after wheat globally and is adversely affected by high heat, which is a significant abiotic stress factor. This study was carried out to determine the physiological and biochemical responses of hybrid corn varieties under heat stress (‘HS’) compared to control (‘C’) conditions during the 2020 and 2021 growing seasons. The experiment was conducted under natural conditions in the Southeastern region of Turkey, where the most intense temperatures are experienced. This experiment used split plots in randomized blocks with three replications, with ‘HS’ and ‘C’ growing conditions applied to the main plots and the different hybrid corn varieties (FAO 650) planted on the sub plots. Mean values of days to 50% tasseling (DT, day), grain yield (GY, kg ha^−1^), leaf water potential (LWP, %), chlorophyll-a (Chl-a, mg g^−1^), cell membrane damage (CMD, %), and total phenol content (TPC, μg g^−1^) were significantly different between years, growing conditions, and hybrid corn varieties. Changes in the climate played a significant role in the differences between the years and growing conditions (GC), while the genetic characteristics of the different corn varieties explained the differences in outcomes between them. The values of DT, GY, LWP, Chl-a, CMD, and TPC ranged from 49.06–53.15 days, 9,173.0–10,807.2 kg ha^−1^, 78.62–83.57%, 6.47–8.62 mg g^−1^, 9.61–13.54%, and 232.36–247.01 μg g^−1^, respectively. Significant correlations were recorded between all the parameters. Positive correlations were observed between all the variables except for CMD. The increased damage to cell membranes under ‘HS’ caused a decrease in the other measured variables, especially GY. In contrast, the GY increased with decreased CMD. CMD was important in determining the stress and tolerance level of corn varieties under ‘HS’ conditions. The GY and other physiological parameters of ADA 17.4 and SYM-307 candidate corn varieties surpassed the control hybrid corn cultivars. The results revealed that the ADA 17.4 and SYM-307 cultivars might have ‘HS’-tolerate genes.

## Introduction

Corn (*Zea mays* L.) is an important summer-growing cereal grain, which is produced around the world as well as in Turkey, and is used for human consumption, feed for different animal groups, and as a raw material for bio-energy (Ge et al., 2012; Bassu et al., 2014). Approximately 1,102,164 billion tons of corn are produced worldwide, annually (FAO, 2020), providing 19.5% of the global calorie demand (Hussain et al., 2019a). In 2020, Turkey produced 6 million tons of corn, meeting 90% of the domestic market demand (TSI, 2021). Heat stress (‘HS’) is one of the most significant abiotic stresses as it causes a decrease in maize yield per unit area in many parts of the world. Global temperature is expected to increase by 1.5 °C due to an increase of greenhouse gases, especially CO_2_ and methane (CH_4_; IPCC, 2021). ‘HS’ significantly impacts the growth of agricultural crops, especially corn production (Chartzoulakis & Bertaki, 2015). Corn supply problems will arise with the simultaneous increase of ‘HS’ and the global population. Achieving optimum grain yield will no longer be possible if abiotic stresses significantly increase (Landman et al., 2018). Therefore, corn breeding programs, especially those focusing on the breeding of ‘HS’-tolerant corn varieties, should be carried out based on physiological and biochemical screening data of corn varieties.

Corn production has increased primarily by expanding agricultural production areas instead of increasing the corn yield per unit area (OECD/FAO, 2020). However, continuing to increase agricultural production areas is not sustainable because of emerging abiotic stress factors and urbanization (Santpoort, 2020). Optimum environmental conditions are needed in order to determine the genetic grain yield potential of different corn varieties. Extreme climate events, such as drought and ‘HS,’ remain a serious threat to the global food supply (FAO, 2018). ‘HS’ shortens the life cycle of corn (Stone, 2001), reduces light interception, increases respiration, and reduces photosynthesis and flexibility of the cell membrane (Crafts-Brander & Salvucci, 2002). Therefore, ‘HS’ severely constrains agricultural productivity, including corn yield (Rezaei et al., 2015). Significant corn grain yield losses have been reported due to temperatures above 35 °C (Tesfaye et al., 2017). Similarly, 26% grain yield loss has been reported under ‘HS’ conditions between 37 °C and 39 °C compared to control conditions (32–34 °C; Chukwudi, Kutu & Mavengahama, 2021). In one study, corn yield decreased linearly with each degree of temperature increase above 30 °C (Lobell et al., 2011), and Zhao et al. (2017) reported an approximate 7.4% loss in corn yield with each degree of temperature increase. Grain yield also significantly decreases when plants face ‘HS’ for a prolonged period of time (Schauberger et al., 2017). One study found that the radiation use efficiency of corn plants decreased significantly under ‘HS’ up to 36 °C, and dry matter accumulation and grain yield decreased due to low active nitrogen and carbon metabolism (Yang et al., 2019). ‘HS’ causes water loss in the leaves through stomatal openings (Sharma et al., 2015). In addition, ‘HS’ also causes a decrease in leaf water content and total water absorption rate, resulting in a serious decrease in grain yield (Ashraf & Hafeez, 2004). Corn grain yield is affected by the genetic characteristics of the seeds used and the conditions of the growing environment (Yang et al., 2020; Inyang et al., 2021). Although ‘HS’ causes a decrease in grain yield, Karmakar et al. (2016) also reported significant differences in the attributes of corn grown under two different temperatures.

The reproductive stage of corn plants between tasseling and silking is the most sensitive stage to temperatures fluctuations, and high temperatures accelerate the life cycle of tasseling (Siebers et al., 2017). Short- or long-term exposure to high temperature stress during corn growth periods (especially during the most critical tasseling stage) causes metabolic and/or morphological changes (Shim, Lee & Lee, 2017). Some corn genotypes mature earlier under ‘HS’ leading to slight yield losses (Adams, Cockshull & Cave, 2001). In addition, exposure to high temperatures adversely affects the gas exchange, leaf water content, photosynthesis activity and chlorophyll-a and b contents of corn plants (Pessarakli, 2007; Waqas et al., 2017). Neiff et al. (2016) also found that temperatures ranging from 33 °C to 36 °C during the pre-and post-flowering periods reduced chlorophyll content and CO_2_ exchange rate (17%), crop growth rate (17–29%), leaf water content (7–25%), and grain yield (10–45%). High ‘HS’ has detrimental impacts on macromolecules, cellular structures and membranes in corn due to the overproduction of reactive oxygen species (Hussain et al., 2018). In addition, ‘HS’ affects nutrient uptake, limits the functioning of various enzymes such as phenolics (Hussain et al., 2019b; Hasanuzzaman et al., 2020), and reduces corn plant cell membrane elasticity (Zhou et al., 2017).

Phytohormones regulate the biochemical processes of plant cells, which are ubiquitous to plant growth under ‘HS’ conditions (Sharma et al., 2019). The cell organelles of corn plants respond quickly to variations in ambient temperature, sometimes causing the alteration of biochemical and molecular pathways in all parts of the cells (Kosová et al., 2018). ‘HS’ conditions trigger a biochemical response in maize plants where phenolic compounds increase to work against oxidative stress in the cells (Ravazzolo et al., 2021). Phenolic compounds are biochemical materials involved in the vital activities of plants that reduce oxidative effects (Halliwell, 2006; Dong et al., 2014). The antioxidant effects and phenolic content of corn plants are higher than in oat, wheat, and rice (Adom & Liu, 2002).

High-tolerance corn lines and varieties should be bred to reduce the negative impacts of ‘HS’ on corn plants (Chukwudi, Kutu & Mavengahama, 2021). A more comprehensive understanding of the physiological and biochemical responses of corn plants to temperature extremes is needed to identify and breed corn varieties that can withstand extreme heat. Furthermore, determining which physiological and biochemical traits are correlated with corn grain yield will help create the selection criteria for future corn variety breeding programs. Therefore, this study was conducted to (i) determine the physiological and biochemical performances of candidate hybrid corn cultivars under natural ‘HS’ and control conditions, (ii) understand the response mechanisms of these corn cultivars, (iii) explain the relationship between GY and other physiological parameters, and (iv) reveal the physiological or biochemical parameters necessary to measure the stress and tolerance level of corn cultivars.

## Materials and Methods

### Experimental site and climate characteristics

The field experiments were conducted at the Talat Demiroren research station of the South Eastern Anatolian Project Agricultural Research Institute, Sanliurfa Province, Turkey (36° 54′ 13″ N, 38° 55′ 03″E, altitude 378 m). The field studies were carried out in the 2020 and 2021 corn growing periods. The soil of the experimental field had a heavy clay textured, granular structure and the infiltration rate was within the optimum range. The organic matter content and pH levels were between 0.81% and 1.11% and 7.55, and 8.11, respectively.

The study area is located in a semi-arid climate with high temperatures and low relative humidity during the summer season. The corn growing periods in both 2020 and 2021 were drier and hotter compared to the historical temperature and precipitation averages. The amount of precipitation and duration of sunshine during the experiments verified that the region has a semi-arid climate close to the aridity line. The climate data of the study area recorded during the corn growing seasons of 2020 and 2021 are provided in Table 1.

**Table 1:** Meteorological data of the study area. Climate data, such as temperature and relative humidity, dramatically affected the performance of hybrid corn varieties.

Months	Mean temperatures (°C)			Mean maximum temperatures			Mean relative humidity (%)			Total rainfall (mm/m^−2^)			Sunshine duration (hour)		
	2020	2021	ALY	2020	2021	ALY	2020	2021	ALY	2020	2021	ALY	2020	2021	ALY
May	22.8	25.7	22.3	31.0	36.7	28.8	47.7	33.0	38.9	0.0	0.0	29.2	10.1	10.5	10.0
June	28.3	28.5	28.1	36.5	38.2	34.7	35.9	33.1	35.1	0.0	0.2	7.3	11.9	12.1	12.1
July	33.2	32.7	32.0	41.6	43.0	38.8	33.4	32.3	33.4	0.0	0.0	4.2	13.0	12.8	12.3
August	30.7	31.3	31.6	40.7	42.0	38.4	34.2	39.3	31.6	0.0	0.0	5.6	12.2	12.6	11.3
September	28.7	30.4	27.2	38.3	40.1	34.0	45.6	38.5	30.2	0.2	0.0	7.6	10.0	10.3	10.0
October	22.1	23.3	20.6	31.8	32.3	27.1	56.2	46.5	43.7	0.0	0.0	31.4	7.1	6.9	7.9
November	13.1	14.0	13.1	19.9	18.9	18.8	70.0	64.2	65.9	76.4	11.5	51.7	6.4	6.6	6.0
December	9.0	11.2	7.6	15.7	17.0	12.0	68.5	65.2	69.9	8.6	16.4	80.2	4.4	5.0	4.2
Average	23.5	24.7	22.8	31.9	33.5	29.1	48.9	44.0	43.6	85.2	28.1	217.2	9.4	9.6	9.2

**Note:**

ALY, Average for long years (1950–2021).

‘HS’ conditions were present between May 1 and September 1, while August 1 to December 1 had ‘C’ conditions, which are the optimum conditions. The ‘HS’ and ‘C’ conditions in the study area were determined using historical climate data. The temperature difference between the ‘HS’ and ‘C’ conditions was 4.77 °C in the first year of the study (2020), and increased to 6.65 °C in the second year (2021). Temperature differences were recorded both between years and corn growing conditions (GC). More extreme climate conditions occurred in the first year of the experiment.

**Figure 1:**
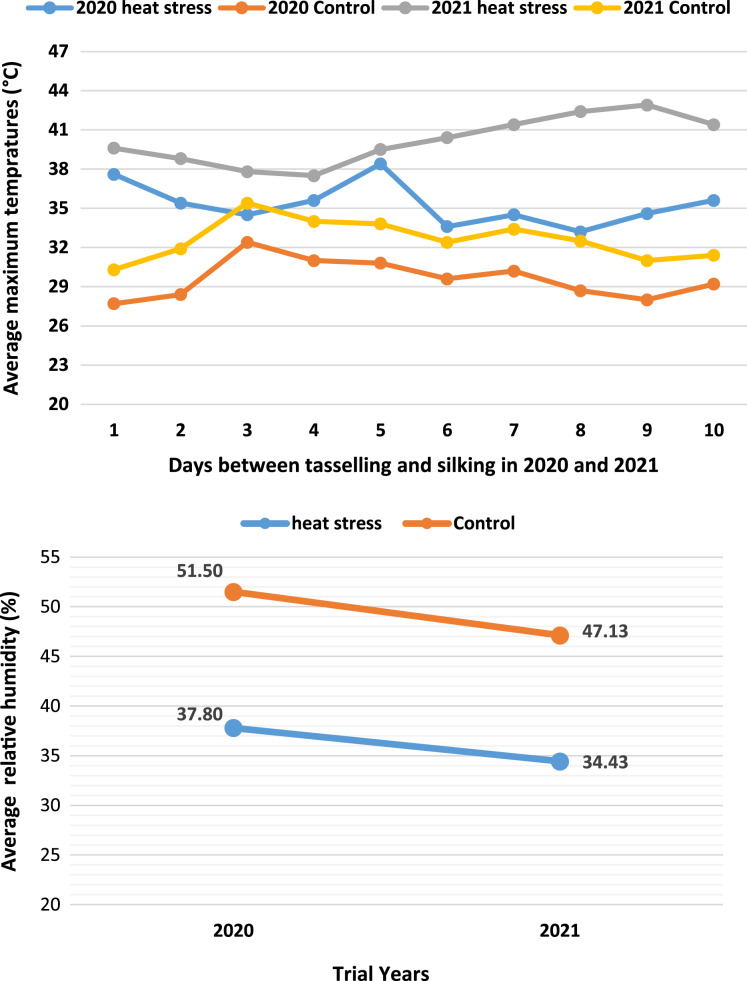
Average relative humidity changes in heat stress (HS) and control (C) conditions and 10-day average maximum temperature values between tasseling and silking in both test years. Relative humidity <40% in HS conditions occurred as a secondary stress to the plants. Pollen fertility was adversely affected in the days between tasseling and silking due to the high-temperatures in HS conditions.

The average relative humidity also differed between the experimental years and the ‘HS’ and ‘C’ conditions. In the first year of the study, the relative humidity during ‘HS’ conditions was 13.7% lower than under ‘C’ conditions, but 12.7% lower in the second year. The mean relative humidity values during ‘HS’ conditions in both experimental years were below 40%. Similar to the temperature and relative humidity values, the daily average maximum temperature values between the tasseling and silking periods during ‘HS’ conditions were higher than those recorded in ‘C’ conditions of both experimental years. The temperature exceeded 40 °C on some days during the 2021 growing season, especially under ‘HS’ conditions (Fig. 1).

### Plant material, experimental design, and cultural practices

Eleven candidate hybrid corn varieties from the national corn breeding program and two commercial hybrid corn varieties, used as controls, were used in this study. All included varieties were classified as having moderate maturity (FAO 650) and are widely grown in ‘HS’ areas and regions of the world. Some traits of the corn varieties used in the study are given in Table 2.

**Table 2:** Characteristics of hybrid maize varieties used in the experiment. Thirteen hybrid maize varieties (eleven from Turkey, two from the USA), with same ear type and colour and FAO maturity group, were used in the study.

Variety name	Ear colour ve type	Source	FAO maturity group
ADA 17.1	Yellow dent	Turkey	650
ADA 17.2	Yellow dent	Turkey	650
ADA 17.3	Yellow dent	Turkey	650
ADA 17.4	Yellow dent	Turkey	650
ADA 17.5	Yellow dent	Turkey	650
ADA 17.6	Yellow dent	Turkey	650
ADA 17.7	Yellow dent	Turkey	650
ADA 17.8	Yellow dent	Turkey	650
SYM-305	Yellow dent	Turkey	650
SYM-306	Yellow dent	Turkey	650
SYM-307	Yellow dent	Turkey	650
Check-1	Yellow dent	USA	650
Check-2	Yellow dent	USA	650

This experiment used split plots in randomized blocks with three replications, with ‘HS’ and ‘C’ growing conditions applied to the main plots and the different corn varieties planted in the sub plots. The inter row and intra row spacings were 70 and 17 cm, respectively. Each plot had four rows, 5 m in length, and the area of each plot was 14 m^2^. Each plot had 115 to 120 corn plants. Fertilizer was applied in the seedbeds at a rate of 350 kg ha^−1^ in the form of diammonium phosphate (18%N-46%P_2_O_5_-0%K_2_O) during sowing (Kucuk, Cevheri & Mutlu, 2019). Nitrogen (350 kg N ha^−1^) was applied as urea (46% N) at the second and fourth weeks of sowing. Considering climate conditions and the water requirements of corn plants, a total of 660 and 600 mm irrigation water was applied in the ‘HS’ and ‘C’ conditions of the first year, respectively, and 710 and 630 mm irrigation water was applied in ‘HS’ and ‘C’ conditions of the second year, respectively, when the ‘HS’ was more serious. Corn cobs were harvested between August 25–29 and December 1–3 in the ‘HS’ and ‘C’ conditions of the first year, respectively, and between August 24–25 and November 28–30 in the second year. The harvest dates differed between the years due to changes in climate, and the cobs were harvested when the corn kernels had 24–25% moisture.

### Data collection

#### Leaf water potential (LWP, %)

The flag leaves of five randomly selected plants in each plot were collected during each of the experiment years between the tasseling and silking phenological periods and then quickly transferred to the laboratory in an ice container. The leaves were cut using a defined template to fit into the Petri dishes, and the wet weight (WW) of the leaf samples was measured using a precision balance. After weighing, the leaves were placed into the petri dishes filled with distilled water, submerging the cutting area, and kept in distilled water for 12 h, when the turgid weight of the leaves (TW) was measured. After determining the WW and TW, the leaves were kept in an oven at 70 °C for 24 h, and the dry weight (DW) was then determined. The leaf water potential (LWP) was calculated using the following equation (Bowman, 1989):

$\rm{LWP \; (\%) = (WW-DW) / (TW-DW)*100}$

#### Chlorophyll – a (Chl-a, mg g^−1^)

Leaf samples were taken and transferred to the laboratory for the Chl-a analysis using the same methods that were used for the LWP analysis. Fresh leaf samples (0.1 g) in each plot were weighed on a precision scale, homogenized with 3 ml 80% (v/v) acetone in a mortar, and poured into Eppendorf tubes placed with white band filter paper and filtered. The filtered suspension was brought to 10 ml with 80% (v/v) acetone. Immediately after vortexing all Eppendorf tubes, the absorbance at 663 nm was determined using a spectrophotometer for Chl-a contents (Arnon, 1949).

Chl-a is a sub-pigment of chlorophyll. In this study, the Chl-a content was calculated using the following equation (Lichtenthaler & Wellburn, 1983):

$\rm{Chlorophyll - a\; (Chl-a, mg \; g^{-1}) = \Delta A \; 663 \times 12.70 - \Delta A\ 645 \times 2.69/mg \;sample \;weight}$

In the equation, *ΔA*663 × 12.70 and *ΔA*645 × 2.69 are optical absorptions in wavelengths determined by the spectrophotometer and multiplied by concentration (mg g^−1^).

#### Cell membrane damage (CMD, %)

After randomly selecting leaves, a 100 mg leaf sample from each selected leaf was weighed and the samples were washed three times with distilled water. The plant samples were transferred to test tubes containing 10 ml of deionized water. The tubes were then capped and kept in a water bath at 32 °C for 2 h. The electrical conductivity (EC) of the solution was measured with an EC meter and an EC1 value was determined. In the second phase, the samples were autoclaved at 121 °C for 20 min to destroy all cells and allow organic and inorganic ions to pass into the solution. After bringing the samples to room temperature, the EC of the solutions was measured, and an EC2 value was determined. The cell membrane damage (CMD, %) value was calculated using the following equation (Sairam, 1994):

$\rm{CMD=[1-(EC1/EC2)] \times 100}$

#### Total phenol content (TPC, μg g^−1^)

Flag leaves of five randomly selected plants were sampled from each plot between the tasseling and silking phenological periods. A 100 mg sample of each of the flag leaf samples was wrapped in aluminum foil and quickly transferred to the laboratory within a nitrogen tank at −198 °C, and then placed in a refrigerator at −20 °C (Azzouzi et al., 2021). A 100 g sample of plant tissues from each sample was then homogenized in 3 mL methanol (80%), and then more 80% methanol was added until the final volume of the homogenate reached 5 mL. From the prepared solutions, 76 µL of sample was taken and diluted with 684 µL of 80% methanol. The final volume of each of the tubes with plant tissues was 760 µL. For both the control samples and test samples, 120 µL of Folin-Ciocalteu reagent (1 N) was pipetted and incubated for 5 min at room temperature. Then, 320 µL of 20% Na_2_CO_3_ was pipetted, 800 µL of deionized water was added, and then the tubes with a final volume of 2,000 µL were vortexed. The absorbance values of the control samples and test plant samples were determined at 765 nm using a spectrophotometer (Singleton, Orthofer & Lamuela-Raventos, 1999).

### Data analysis

A statistical analysis was carried out using JUMP (Version 13.2.0) statistical software. The differences in the traits between the growing conditions were tested using an analysis of variance (ANOVA). A statistical analysis was carried out for each experimental year, separately, as well as the 2-year combined data. The degrees of freedom and F ratio for all parameters were calculated in the combined-year data. The least significant difference (LSD) multiple comparison test, set at a 95% probability, was used where ANOVA indicated significant differences between the mean values for year, the growing conditions, and the plant varieties. The relationships between all traits examined in the study were determined using the Spearman’s correlation test.

## Results

The difference in all parameters between year, growing conditions (GC), and corn varieties was statistically significant (*P* ≤ 0.01). The effect of GCxV interaction on DT, LWP, Chl-a and CMD was significant at the *P* ≤ 0.01 level, and it was significant for TPC at the *P* ≤ 0.05 level. In contrast, the interaction of GCxV had no significant effect on GY. The F ratio and degrees of freedom of variance for all parameters in the combined-year data are shown in Table 3.

**Table 3:** Combined-year F ratios and degrees of freedom for each of the measured traits. The combined-year F ratio, DF, CV, means, and level of probability values of each of the measured traits were calculated using the JUMP statistical software.

Source	DF	F ratio					
		DT	GY	LWP	Chl-a	CMD	TPC
Y	1	122.658**	36.404**	113.664**	50.823**	113.565**	101.422**
R (Y)	4	4.224	0.601	3.345	0.858	2.604	5.994
GC	1	398.412**	127.874**	436.368**	197.986**	419.329**	270.099**
Y*GC	1	41.536**	0.035^ns^	0.167^ns^	0.006^ns^	0.022^ns^	8.958**
R*GC*Y	4	0.685	1.445	0.692	1.645	1.065	0.460
V	12	26.682**	60.344**	63.496**	35.901**	72.430**	92.354**
Y*V	12	1.625^ns^	0.327^ns^	0.996^ns^	0.943^ns^	1.165^ns^	0.714^ns^
GC*V	12	4.908**	1.346^ns^	2.149**	4.749**	7.277**	2.053*
Y*GC*V	12	1.022^ns^	0.465^ns^	2.753**	0.856^ns^	1.798*	1.305^ns^
Error	96						
CV(%)		2.50	9.03	1.83	12.63	10.34	2.32
Mean		51.11	9,990.1	81.09	7.55	11.57	239.68

**Notes:**

* Significant at 0.05 level of probability.

** Significant at 0.01 level of probability.

ns, not significant; DF, degrees of freedom; CV, coefficient of variation; R, repetition; Y, year; GC, growth condition; V, Variety.

Agronomic traits such as DT and GY significantly differed between year, GC, and corn varieties (Table 4). The highest DT among all growing conditions and plant varieties was 52.24 days in the first year of the study, while the highest DT value in the second year decreased by an average of 2 days to 49.97 days. The combined-year data indicated that the corn varieties flowered (tasseling) an average of 53.15 days under ‘C’ conditions, and an average of 49.06 days under ‘HS’ conditions. The DT levels in the ‘HS’ conditions of 2021 were lower than during the ‘C’ conditions of 2020. The ADA 17.3 corn variety stood out as the earliest variety in the experiment, and the SYM-307 corn variety was the latest variety. One of the most important findings of the study was recorded for the GY. The mean GY value in the first year was 10,426.09 kg ha^−1^, but this value decreased by 8.36% in 2021. The GY value in ‘HS’ conditions was 15.12% lower than in the ‘C’ conditions. The GY values of SYM-307 (12,608.9 kg ha^−1^) and ADA 17.4 (12,361.5 kg ha^−1^) candidate cultivars were higher than the control hybrid maize cultivars.

**Table 4:** The effect of different growth conditions on the DT and GY of hybrid maize varieties. The performances (DT and GY) of hybrid corn varieties under growing conditions in 2020 and 2021 were compared.

Days to 50% tassel (day)									
**Years**	**2020**			**2021**			**202–2021**		
**GC/V**	**HS**	**C**	**Mean**	**HS**	**C**	**Mean**	**HS**	**C**	**Mean**
ADA 17.1	48.33^jkl^	55.67^bcd^	52.00^cd^	46.67	49.00	47.83^ef^	47.50^l^	52.33^efg^	49.92^d^
ADA 17.2	47.33^l^	56.00^bc^	51.67^d^	47.00	52.00	49.50^de^	47.17^l^	54.00^bcd^	50.58^cd^
ADA 17.3	44.33^m^	54.00^de^	49.17^f^	44.00	50.00	47.00^f^	44.17^m^	52.00^e-h^	48.08^f^
ADA 17.4	50.00^hıj^	53.00^ef^	51.50^d^	49.33	51.67	50.50^bcd^	49.67^jk^	52.33^efg^	51.00^c^
ADA 17.5	52.00^fg^	55.00^cd^	53.50^b^	51.00	53.33	52.17^ab^	51.50^f-ı^	54.17^bc^	52.83^b^
ADA 17.6	50.33^ghı^	57.00^ab^	53.67^b^	50.67	53.67	52.17^ab^	50.50^ıj^	55.33^ab^	52.92^b^
ADA 17.7	48.00^kl^	52.00^fg^	50.00^ef^	47.67	50.67	49.17^de^	47.83^l^	51.33^f-ı^	49.58^de^
ADA 17.8	51.33^fgh^	57.00^ab^	54.17^b^	50.00	52.67	51.33^bc^	50.67^hıj^	54.83^ab^	52.75^b^
SYM-305	49.33^ıjk^	53.00^ef^	51.17^de^	47.33	48.67	48.00^ef^	48.33^kl^	50.83^hıj^	49.58^de^
SYM-306	48.00^kl^	52.00^fg^	50.00^ef^	46.33	48.33	47.33^f^	47.17^l^	50.17^ıj^	48.67^ef^
SYM-307	53.00^ef^	58.00^a^	55.50^a^	52.33	54.33	53.33^a^	52.67^def^	56.17^a^	54.42^a^
Check-1	50.00^hıj^	56.00^bc^	53.00^bc^	49.33	50.67	50.00^cd^	49.67^jk^	53.33^cde^	51.50^c^
Check-2	52.00^fg^	55.67^bcd^	53.83^b^	50.00	52.67	51.33^bc^	51.00^g-j^	54.17^bc^	52.58^b^
Mean ± SE r = 3	49.54 ± 0.65^b^	54.95 ± 0.54^a^	52.24 ± 0.52^A^	48.59 ± 0.64^b^	51.36 ± 0.55^a^	49.97 ± 0.57^B^	49.06 ± 0.63^b^	53.15 ± 0.50^a^	51.11
CV (%)	2.10			2.88			2.50		
LSD (0.05)	GC: 0.50** V: 1.27** GCxV: 1.80**			GC: 0.66** V: 1.67** GCxV: ns			Y: 0.41** GC: 0.41** V:1.04** GCxV: 1.47**		
**Grain yield (kg ha^−1^)**									
ADA 17.1	9,364.7	10,982.3	10,173.5^cd^	9,046.3	10,313.0	9,679.7^cd^	9,205.5	10,647.7	9,926.6^d^
ADA 17.2	10,167.3	11,384.9	10,776.1^bc^	9,769.7	10,856.0	10,312.8^cd^	9,968.5	11,120.5	10,544.5^bcd^
ADA 17.3	9,738.0	11,560.5	10,649.2^bc^	8,259.0	11,280.0	9,769.5^cd^	8,998.5	11,420.2	10,209.4^cd^
ADA 17.4	11,483.5	14,400.7	12,942.1^a^	10,399.3	13,162.4	11,780.9^a^	10,941.4	13,781.5	12,361.5^a^
ADA 17.5	10,369.0	12,146.8	11,257.9^bc^	9,829.3	11,022.0	10,425.7^cd^	10,099.2	11,584.4	10,841.8^bc^
ADA 17.6	10,668.0	11,367.9	11,017.0^bc^	8,372.1	10,671.3	9,521.7^d^	9,520.0	11,019.6	10,269.8^bcd^
ADA 17.7	8,709.3	10,082.3	9,395.8^de^	8,004.0	8,417.7	8,210.8^e^	8,356.7	9,250.0	8,803.3^e^
ADA 17.8	8,528.7	9,347.9	8,938.3^e^	7,937.0	8,678.0	8,307.5^e^	8,232.8	9,012.9	8,622.9^e^
SYM-305	5,969.4	8,503.0	7,236.2^f^	5,613.7	7,517.4	6,565.5^f^	5,791.6	8,010.2	6,900.9^f^
SYM-306	5,276.1	7,256.9	6,266.5^f^	4,282.3	6,349.7	5,316.0^g^	4,779.2	6,803.3	5,791.2^g^
SYM-307	12,231.7	13,636.2	12,933.9^a^	11,636.3	12,931.3	12,283.8^a^	11,934.0	13,283.8	12,608.9^a^
Check-1	10,380.4	12,464.2	11,422.3^b^	9,752.3	11,337.0	10,544.7^bc^	10,066.4	11,900.6	10,983.5^b^
Check-2	11,855.5	13,203.1	12,529.3^a^	10,855.0	12,115.3	11,485.2^ab^	11,355.3	12,659.2	12,007.2^a^
Mean ± SE r = 3	9,595.5 ± 578.9^b^	11,256.67 ± 569.2^a^	10,426.1 ± 567.4^A^	8,750.5 ± 568.0^b^	10,357.8 ± 572.1^a^	9,554.1 ± 559.8^B^	9,173.0 ± 568.4^b^	10,807.2 ± 567.4^a^	9,990.1
CV (%)	9.09			8.95			9.03		
LSD (0.05)	GC: 431.31** V: 1099.63** GCxV: ns			GC: 389.48** V: 992.99** GCxV: ns			Y: 286.86** GC: 286.86** V: 731.36** GCxV: ns		

**Notes:**

** Significant at 0.01 level of probability.

HS, heat stress condition; C, control condition; GC, growth condition; V, variety; r, replications; SE, Statistical Error; ns, not significant; CV, coefficient of variation.

The means indicated with the same letter in the same column and row are not significantly different according to the JUMP test at *P* ≤ 0.05.

Leaf water potential (LWP) is an important physiological parameter. The LWP value was 82.35% in the first year, and decreased by 3.06% in the second year. The LWP value also decreased by 5.92% in ‘HS’ conditions compared to ‘C’ conditions. The Check-2 corn variety had the highest LWP value (86.30%), and the SYM-307 and ADA 17.4 varieties, which stood out in terms of GY, were placed in a sub-statistical group (LWP, 84%). Chl-a was analyzed for year, corn variety, and GC. The Chl-a sub-pigment value was 8.09 mg g^−1^ in the first year, while this value decreased by approximately 1 mg g^−1^ in the second year. The combined-year average Chl-a value of the corn varieties was 6.47 mg g^−1^ in ‘HS’ conditions, which increased to 8.62 mg g^−1^ in ‘C’ conditions. The Chl-a pigment decreased by 24.94% in ‘HS’ conditions compared to ‘C’ conditions. The ADA 17.4 candidate variety also had the highest Chl-a value (Table 5).

**Table 5:** The effect of different growth conditions on the physiological traits of hybrid maize varieties. The Chl-a and LWP of hybrid corn varieties significantly differed between experiment years and growing conditions.

Leaf water potential (%)									
**Years**	**2020**			**2021**			**2020–2021**		
**GC/V**	**HS**	**C**	**Mean**	**HS**	**C**	**Mean**	**HS**	**C**	**Mean**
ADA 17.1	77.01	83.44	80.23^ef^	75.99^ı^	79.52^fg^	77.76^d^	76.50^mn^	81.48^e-h^	78.99^f^
ADA 17.2	78.74	85.39	82.06^d^	75.59^ıj^	81.30^ef^	78.45^d^	77.17^lmn^	83.35^bcd^	80.26^e^
ADA 17.3	79.74	85.37	82.56^d^	77.45^ghı^	80.29^f^	78.87^cd^	78.59^jkl^	82.83^cde^	80.71^de^
ADA 17.4	81.88	88.78	85.33^bc^	80.30^f^	85.96^bc^	83.13^a^	81.09^f-ı^	87.37^a^	84.23^b^
ADA 17.5	81.22	85.91	83.57^cd^	79.33^fgh^	83.63^d^	81.48^b^	80.28^g-j^	84.77^b^	82.53^c^
ADA 17.6	80.37	84.03	82.20^d^	79.51^fg^	82.89^de^	81.20^b^	79.94^hıj^	83.46^bc^	81.70^cd^
ADA 17.7	78.97	84.96	81.96^de^	75.95^ı^	84.26^cd^	80.10^bc^	77.46^lm^	84.61^b^	81.03^de^
ADA 17.8	74.85	82.37	78.61^fg^	73.34^jk^	76.53^ı^	74.93^e^	74.09^op^	79.45^ıjk^	76.77^g^
SYM-305	73.70	79.03	76.37^h^	72.34^k^	77.10^hı^	74.72^e^	73.02^p^	78.07^klm^	75.54^h^
SYM-306	75.96	79.51	77.73^gh^	75.25^ıj^	76.23^ı^	75.74^e^	75.61^no^	77.87^klm^	76.74^gh^
SYM-307	84.48	87.99	86.24^ab^	80.89^ef^	86.30^abc^	83.60^a^	82.69^c-f^	87.15^a^	84.92^b^
Check-1	84.15	87.40	85.78^b^	79.30^fgh^	87.04^ab^	83.17^a^	81.72^d-g^	87.22^a^	84.47^b^
Check-2	86.74	89.23	87.99^a^	80.96^ef^	88.26^a^	84.61^a^	83.85^bc^	88.75^a^	86.30^a^
Mean ± SE r = 3	79.83 ± 1.08^b^	84.88 ± 0.89^a^	82.35 ± 0.96^A^	77.40 ± 0.79^b^	82.26 ± 1.14^a^	79.83 ± 0.93^B^	78.62 ± 0.92^b^	83.57 ± 0.99^a^	81.09
CV (%)	1.91			1.73			1.83		
LSD (0.05)	GC: 0.72** V: 1.83** GCxV: ns			GC: 0.63** V: 1.60** GCxV: 2.26**			Y: 0.47** GC: 0.47** V: 1.20** GCxV: 1.70**		
**Chlorophyll-a (mg g^−1^)**									
ADA 17.1	7.09^ı-l^	8.28^f-ı^	7.69^c^	6.01	7.48	6.74^cd^	6.55^hıj^	7.88^d-g^	7.21^de^
ADA 17.2	8.12^ghı^	12.26^a^	10.19^b^	6.73	10.19	8.46^b^	7.43^fgh^	11.22^a^	9.33^b^
ADA 17.3	6.50^klm^	6.71^j-m^	6.61^d^	4.91	5.67	5.29^ef^	5.71^j^	6.19^ıj^	5.95^f^
ADA 17.4	10.59^bc^	11.96^ab^	11.27^a^	8.65	11.53	10.09^a^	9.62^b^	11.7^a^	10.68^a^
ADA 17.5	6.37^lm^	8.39^f-ı^	7.38^cd^	5.80	8.41	7.11^cd^	6.08^ıj^	8.40^c-f^	7.24^de^
ADA 17.6	5.66^mn^	8.83^e-h^	7.24^cd^	5.33	7.62	6.48^de^	5.49^j^	8.23^c-g^	6.86^e^
ADA 17.7	6.14l^mn^	10.23^cd^	8.18^c^	5.88	8.23	7.05^cd^	6.01^j^	9.23^bc^	7.62^de^
ADA 17.8	6.14l^mn^	9.31^c-g^	7.73^c^	5.28	8.91	7.10^cd^	5.71^j^	9.11^bc^	7.41^de^
SYM-305	4.81^n^	8.04^g-j^	6.43^d^	3.74	6.27	5.01^f^	4.28^k^	7.16^ghı^	5.72^f^
SYM-306	3.26^o^	7.25^ı-l^	5.26^e^	2.33	5.09	3.71^g^	2.80^l^	6.17^ıj^	4.48^g^
SYM-307	9.59^c-f^	9.92^cde^	9.75^b^	7.58	9.35	8.46^b^	8.58^b-e^	9.63^b^	9.11^b^
Check-1	7.84^h-k^	8.14^ghı^	7.99^c^	7.29	8.53	7.91^bc^	7.57^e-h^	8.34^c-f^	7.95^cd^
Check-2	9.21^d-h^	9.77^cde^	9.49^b^	7.48	7.81	7.64^bcd^	8.34^c-f^	8.79^bcd^	8.57^bc^
Mean ± SE r = 3	7.02 ± 0.56^b^	9.16 ± 0.45^a^	8.09 ± 0.46^A^	5.92 ± 0.47^b^	8.08 ± 0.49^a^	7.00 ± 0.46^B^	6.47 ± 0.51^b^	8.62 ± 0.45^a^	7.55
CV (%)	10.33			15.10			12.63		
LSD (0.05)	GC: 0.38** V: 0.97** GCxV: 1.37**			GC: 0.48** V: 1.23** GCxV: ns			Y: 0.30** GC: 0.30** V: 0.77** GCxV: 1.09**		

**Notes:**

** Significant at 0.01 level of probability.

HS, heat stress condition; C, control condition; GC, growth condition; V, variety; r, replications; SE, Statistical Error; ns, not significant; CV, coefficient of variation.

The means indicated with the same letter in the same column and row are not significantly different according to the JUMP test at *P* ≤ 0.05.

The cell membrane damage (CMD) value was significantly different between year, corn variety, and GC. Cell membrane damage was 12.60% in 2021, and 10.55% in 2020 when the growing conditions were better. The membrane damage was 9.61% in ‘C’ conditions and increased to 13.54% in ‘HS’ conditions. The lowest CMD values were obtained in the ADA 17.4 and SYM-307 candidate cultivars. The difference between the lowest and the highest CMD values was nearly two-fold. The TPC value in 2020 was 244.17 μg g^−1^, and a 3.67% decrease was recorded in 2021. The difference in TPC value between ‘HS’ and ‘C’ conditions was even higher. The mean TPC value of corn cultivars in ‘C’ conditions was 247.01 μg g^−1^; the TPC value decreased by 5.93% in ‘HS’ conditions. Candidate cultivar ADA 17.4 had the highest combined-year TPC value, indicating that it is genetically tolerant to stress conditions. In addition, the TPC difference between the ‘HS’ and ‘C’ conditions in the ADA 17.4 corn cultivar was 3.62%, while the differences in Check-1 and Check-2 cultivars between the two conditions were 7.18% and 6.17%, respectively. These results showed that the ADA 17.4 candidate corn cultivar maintains its stability between optimum and stress conditions (Table 6).

**Table 6:** The effect of different growth conditions on the biochemical traits of hybrid maize varieties. The biochemical traits of hybrid maize cultivars, such as CMD and TPC, differed under ‘HS’ and ‘C’ growth conditions.

Cell membrane damage (%)									
**Years**	**2020**			**2021**			**2020–2021**		
**GC/V**	**HS**	**C**	**Mean**	**HS**	**C**	**Mean**	**HS**	**C**	**Mean**
ADA 17.1	8.77^h-l^	7.10^lmn^	7.94^efg^	11.66^hıj^	8.23^nop^	9.95^fg^	10.22^hıj^	7.67^lmn^	8.94^f^
ADA 17.2	10.66^fgh^	8.46^jkl^	9.56^cd^	13.21^fgh^	9.48^lmn^	11.35^e^	11.94^fg^	8.97^jkl^	10.46^e^
ADA 17.3	10.32^g-k^	6.30^mno^	8.31^d-g^	11.44^h-k^	8.59^m-p^	10.02^fg^	10.88^gh^	7.44^mn^	9.16^f^
ADA 17.4	8.25^klm^	6.21^mno^	7.23^g^	9.15^mno^	7.32^**p**^	8.24^**h**^	8.70^klm^	6.77^**n**^	**7.73** ^**g**^
ADA 17.5	13.62^bcd^	10.68^fgh^	12.15^b^	15.93^cde^	12.22^hı^	14.08^c^	14.78^c^	11.45^gh^	13.11^c^
ADA 17.6	12.48^def^	9.43^g-k^	10.95^bc^	14.33^efg^	11.48^h-k^	12.90^cd^	13.40^de^	10.45^hı^	11.93^d^
ADA 17.7	13.25^cde^	8.50^ı-l^	10.88^bc^	16.48^cd^	10.21^j-m^	13.35^c^	14.87^c^	9.36^ıjk^	12.11^d^
ADA 17.8	15.18^bc^	13.57^bcd^	14.37^**a**^	19.21^b^	14.33^efg^	16.77^b^	17.20^b^	13.95^cde^	15.57^b^
SYM-305	19.37^**a**^	10.59^f-ı^	14.98^**a**^	22.67^**a**^	15.18^de^	18.93^**a**^	21.02^a^	12.89^ef^	**16.95** ^**a**^
SYM-306	15.51^b^	14.06^bcd^	14.79^**a**^	17.40^c^	14.68^def^	16.04^b^	16.46^b^	14.37^cd^	15.41^b^
SYM-307	10.39^f-j^	5.21^no^	7.80^fg^	11.19^ı-l^	7.45^op^	9.32g^h^	10.79^gh^	6.33^**n**^	8.56^fg^
Check-1	11.41^efg^	7.22^lmn^	9.31^de^	12.55^ghı^	9.72^k-n^	11.14^ef^	11.98^fg^	8.47^klm^	10.23^e^
Check-2	13.30^cde^	4.56^o^	8.93^def^	14.20^efg^	9.15^mno^	11.68^de^	13.75^cde^	6.86^n^	10.30^e^
Mean ± SE r = 3	12.50 ± 0.84^a^	8.61 ± 0.82^b^	10.55 ± 0.76^B^	14.57 ± 1.03^a^	10.62 ± 0.75^b^	12.60 ± 0.87^A^	13.54 ± 0.93^a^	9.61 ± 0.77^b^	11.57
CV(%)	12.16			8.76			10.34		
LSD(0.05)	GC: 0.58** V: 1.49** GCxV: 2.11**			GC: 0.50** V: 1.28** GCxV:1.81**			Y: 0.38** GC: 0.38** V: 0.97** GCxV:1.37**		
**Total phenol content (μg g** ^**−1**^ **)**									
ADA 17.1	234.26	240.25	237.26^e^	226.41^hıj^	237.56^efg^	231.99^de^	230.33^klm^	238.91^ıj^	234.62^e^
ADA 17.2	236.63	252.38	244.51^d^	229.70^ghı^	241.52^def^	235.61^d^	233.17^jkl^	246.95^gh^	240.06^d^
ADA 17.3	240.88	258.55	249.72^cd^	220.65^ıjk^	250.22^cd^	235.44^d^	230.77^klm^	254.39^def^	242.58^d^
ADA 17.4	261.79	274.02	267.91^**a**^	258.40^abc^	265.74^**a**^	262.07^**a**^	260.10^bcd^	269.88^a^	264.99^**a**^
ADA 17.5	255.67	270.99	263.33^ab^	245.70^de^	261.67^**a**^	253.69^b^	250.69^fg^	266.33^ab^	258.51^b^
ADA 17.6	226.52	240.71	233.62^ef^	214.52^kl^	231.48^gh^	223.00^f^	220.52^o^	236.09^ıjk^	228.31^fg^
ADA 17.7	230.72	239.73	235.23^ef^	218.32^jkl^	235.70^fgh^	227.01^ef^	224.52^mno^	237.72^ıj^	231.12^ef^
ADA 17.8	226.52	233.40	229.96^fg^	211.28^kl^	229.22^ghı^	220.25^fg^	218.90^op^	231.31^kl^	225.11^g^
SYM-305	216.07	225.88	220.98^h^	210.02^l^	220.74^ıjk^	215.38^gh^	213.04^pq^	223.31^no^	218.18^h^
SYM-306	217.07	231.56	224.31^gh^	196.60^m^	226.30^hıj^	211.45^h^	206.83^q^	228.93l^mn^	217.88^h^
SYM-307	256.82	266.04	261.43^b^	247.41^de^	260.34^ab^	253.88^b^	252.11^efg^	263.19^bc^	257.65^b^
Check-1	247.56	256.54	252.05^c^	230.37^ghı^	258.37^abc^	244.37^c^	238.97^ıj^	257.46^cde^	248.21^c^
Check-2	245.85	262.01	253.93^c^	235.70^fgh^	251.22^bcd^	243.46^c^	240.78^hı^	256.62^def^	248.70^c^
Mean ± SE r = 3	238.18 ± 4.12^b^	250.16 ± 4.40^a^	244.17 ± 4.23^A^	226.54 ± 4.79^b^	243.85 ± 4.16^a^	235.20 ± 4.37^B^	232.36 ± 4.41^b^	247.01 ± 4.25^a^	239.68
CV(%)	2.08			2.56			2.32		
LSD(0.05)	GC: 2.31** V: 5.89** GCxV: ns			GC: 2.74** V: 6.98** GCxV: 9.87**			Y: 1.77** GC: 1.77** V:4.51** GCxV: 6.38*		

**Notes:**

* Significant at 0.05 level of probability.

** Significant at 0.01 level of probability.

HS, heat stress condition; C, control condition; GC, growth condition; V, variety; r, replications; SE, Statistical Error; ns, not significant; CV, coefficient of variation.

The means indicated with the same letter in the same column and row are not significantly different according to the JUMP test at *P* ≤ 0.05.

### Correlations

The lowest and highest correlation coefficients and significance levels of the traits are shown in Table 7. Significant (*P* ≤ 0.01) positive correlations were recorded between GY and TPC (r = 0.83), LWP (r = 0.78), Chl-a (r = 0.71), DT (r = 0.54), LWP and TPC (r = 0.77), Chl-a (r = 0.77), DT (r = 0.65), DT and Chl-a (r = 0.57), TPC (r = 0.53), and Chl-a and TPC (r = 0.67). Significant (*P* ≤ 0.01) negative correlations were observed between CMD and GY (r = −0.72), LWP (r = −0.71), TPC (r = −0.71), Chl-a (r = −0.64), and DT (r = −0.43). Damage to cell membranes caused a decrease in GY, TPC, and other physiological parameters. The lowest correlation coefficient was obtained between CMD and DT parameters, while the highest correlation coefficients were recorded between GY and TPC and LWP.

**Table 7:** Correlation analysis between the traits investigated in the study. High correlation coefficients and significant relationships were observed between grain yield and several physiological and biochemical parameters.

Traits	By traits	Correlation	Count	Lower 95%	Upper 95%	Signif prob (P)	Correlation levels
Chl-a	TPC	0.6697	156	0.5728	0.7481	<0.0001**	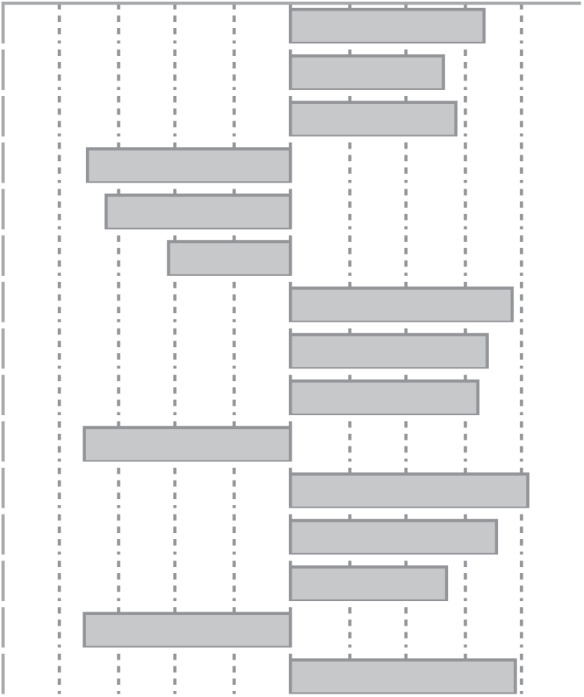
DT	TPC	0.5302	156	0.4069	0.6344	<0.0001**	
DT	Chl-a	0.5712	156	0.4549	0.6683	<0.0001**	
CMD	TPC	−0.7064	156	−0.7773	−0.6178	<0.0001**	
CMD	Chl-a	−0.6383	156	−0.7229	−0.5348	<0.0001*	
CMD	DT	−0.4254	156	−0.5460	−0.2875	<0.0001**	
LWP	TPC	0.7723	156	0.7001	0.8288	<0.0001**	
LWP	Chl-a	0.6768	156	0.5815	0.7537	<0.0001**	
LWP	DT	0.6498	156	0.5487	0.7322	<0.0001**	
LWP	CMD	−0.7132	156	−0.7826	−0.6262	<0.0001**	
GY	TPC	0.8263	156	0.7690	0.8704	<0.0001**	
GY	Chl-a	0.7120	156	0.6247	0.7816	<0.0001**	
GY	DT	0.5441	156	0.4232	0.6460	<0.0001**	
GY	CMD	−0.7163	156	−0.7850	−0.6300	<0.0001**	
GY	LWP	0.7794	156	0.7092	0.8344	<0.0001**	

**Notes:**

* Significant at 0.05 level of probability.

** Significant at 0.01 level of probability.

LWP, Leaf water potential (%); GY, Grain yield (kg ha^−1^); Chl-a: Chlorophyll-a (mg g^−1^); DT, Days to 50% tassel (day); CMD, Cell membrane damage (%); TPC, Total phenol content (μg g^−1^).

## Discussion

### Days to 50% tassel (day) and grain yield (kg ha^−1^)

The decrease in DT values during ‘HS’ may be attributed to the way the plants responded to the adverse environmental conditions; in order to produce grain under such harsh conditions, the plants may have accelerated their life cycles and tasseling stages (Wang et al., 2022). The disruptions to protein and chlorophyll synthesis, insufficient gas exchange through stomatal openings, and water loss through excessive leaf transpiration likely triggered early flowering (tasseling). Early flowering in the maize plants caused a decrease in other physiological parameters, especially GY. The stressed plants could not sufficiently complete their vegetative and morphological processes and experienced some problems at cellular level. Moderate correlations were seen between DT and other physiological parameters, especially GY. Similarly, Cao et al. (2008) observed that early flowering accelerated the tasseling process in maize when the temperature was above 35 °C. The male and female organs of the plants are damaged under ‘HS’ conditions. In addition, pollen germination, pollen tube growth, and viability of the ovules are inhibited, sterility is significantly increased, and grain yield is decreased with the increased ‘HS’ conditions (Maheswari et al., 2012). Lobell et al. (2011) found that a slight increase in temperature during flowering may cause a significant decrease in GY. In ‘HS’ conditions, the pollen fertility of maize plants likely decreases the tasseling period, which is the most sensitive period, disrupting the synchronization between tasseling and silking, resulting in decreased GY.

Previous studies have shown that both environmental conditions and the genetic characteristics of cultivars impact GY (Gao et al., 2021; Liu et al., 2021; Han et al., 2022). Environmental factors, such as high temperature and low relative humidity, adversely affect plant growth leading to yield losses because of the amount of water lost through evapotranspiration. Pollen viability decreases when daily temperatures are 40 °C and above during the reproductive stage (between tasseling and silking), decreasing GY. A correlation analysis indicated that an increase in physiological parameters such as LWP and Chl-a also increased GY, while an increase in CMD decreased GY. ‘HS’ conditions caused damage at the cellular level, deteriorated protein synthesis, disrupted photosynthesis and respiration, and decreased the carbohydrate reserves of the plants, chlorophyll amounts, and the rate of photosynthesis. Because of these changes, the plants could not grow properly, and GY significantly decreased. Yang et al. (2019) observed similar adverse effects on the protein and starch metabolism in leaves under serious ‘HS’ conditions leading to decreases in yield and quality. These GY decreases due to ‘HS’ conditions are also in line with the findings of Cairns et al. (2013). Similarly, Yang et al. (2020) reported a significant decrease in dry matter accumulation, tasseling day, chlorophyll content, and GY in maize under ‘HS’ conditions. The GY loss (22.9%) reported by Yang et al. (2020) was close to the GY loss recorded in this study (15%). High temperatures cause the denaturation of the chlorophyll sub-pigments involved in photosynthesis (Waraich et al., 2012; Mutlu, 2021) and deteriorate other major physiological processes (Mangani et al., 2019), shortening the grain filling time, and reducing the GY. In parallel with these findings, Siebers et al. (2017) found that temperatures over 35 °C inhibited ovary fertilization and the grain filling process of corn plants.

### Leaf water potential (%) and Chlorophyll-a (mg g^−1^)

The leaves are the plant organelles most affected by stress conditions (Killi et al., 2017), and they provide important information about the level of plant stress (Li et al., 2020). High temperatures cause water loss in the leaves, decrease the fluid of the cytoplasm organelle in plant cells, and decrease the flexibility of the cell membranes (Young, Wilen & Bonham-Smith, 2004; Li & Howell, 2021). The chloroplast organelles are also adversely affected by ‘HS’ conditions (Li & Zhang, 2022). Plant leaves wilt, shrink, and curl inward when the stress level is higher (Butler, Mueller & Huybers, 2018). Despite these protective reflexes, the water carried from the roots cannot compensate for the amount of water lost by the transpiration in the leaves (Zhu et al., 2019). Significant negative correlations were observed between LWP and CMD, while significant positive correlations were recorded between the remaining parameters. Flexibility of the cell membranes decreases under ‘HS’ conditions. An increase in ‘HS’ increases cell damage and electrolyte leakage, adversely affecting LWP. The water in the leaf, which is important to both food production and photosynthesis, also indirectly affects the GY of corn varieties. Alam et al. (2017) also found that ‘HS’ decreased leaf water content and increased leaf senescence, resulting in significant maize yield loss.

Chl-a pigments are also adversely affected by stress and undergo structural deterioration (Karim, Fracheboud & Stamp, 1999; Mohammed & Tarpley, 2010). Therefore, in addition to the defects in the photosynthesis mechanism seen with stress, leaves cannot sufficiently absorb the light energy to reduce carbon dioxide, sugar, and other plant substances. Sensitive corn varieties cannot tolerate high ‘HS.’ The formation of reactive oxygen species such as O_2_, OH and H_2_O_2_ in the cells of sensitive corn varieties causes damage to organelles such as chloroplasts and mitochondria in the plant cells (Guo, Zhou & Zhang, 2006). In parallel with these findings, many researchers have reported less photosynthesis and less assimilate accumulation in the leaves, shortened grain filling periods, and decreased corn grain yield due to dry matter loss under ‘HS’ conditions (Yan et al., 2017; Baiyeri et al., 2019). Low chlorophyll sub-pigments such as chlorophyll-a and b formation under ‘HS’ conditions also lowers GY (Yamori, Hikosaka & Way, 2014). Consistent with this study, Marchand et al. (2005) reported that the activity of photosystem II was significantly reduced or stopped under ‘HS,’ which disrupted the functioning of photosynthetic pigments such as chl-a and b.

### Cell membrane damage (%) and total phenol content (µg g^−1^)

The CMD is the most practical and accurate indicator of the plant’s stress level at the cellular level. When the temperature rises significantly, reactive oxygen species accumulate in the cells of sensitive corn varieties, and these harmful oxygen species cause peroxidation in the membranes. A significant negative correlation was recorded between CMD and other parameters. Without water, the membranes lose flexibility and are damaged over time. Zhang et al. (2005) also reported that ‘HS’ increased membrane permeability and damaged mesophyll cells. In addition, decreases in other physiological and morphological characteristics, especially GY, were reported as the damage rate of membranes increased (Zhang et al., 2005). In contrast, resistant corn varieties have tolerance mechanisms such as the ability to make ionic and osmotic adjustments, membrane permeability adjustments, and these varieties can even protect and repair the proteins and cell membranes damaged by ‘HS’ (Vinocur & Altman, 2005).

The TPC accumulates more in plant tissues up to a certain stress level in ‘HS’ compared to ‘C’ conditions. The photosynthesis and gas exchange in the leaves were disrupted during ‘HS’ conditions due to a decrease in Chl-a pigment. The phenolic sub-pigments deteriorated and decreased accordingly. Environmental factors and the characteristics of the corn variety both significantly affect TPC. Intense ‘HS’ may adversely affect the structure of enzymes such as antioxidants and phenols, leaving plants vulnerable (Yamakawa et al., 2007). Consistent with these findings, Shahzad et al. (2011) reported that the total phenol content of corn plants decreased under high-temperature conditions.

## Conclusions

The results of this study revealed that some physiological and biochemical reactions in corn leaves under stress conditions impact GY. High correlation coefficients were observed between GY and LWP, Chl-a, CMD, and TPC. The high temperatures and dry weather conditions experienced during 2021 ‘HS’ conditions compared to the ‘C’ conditions of 2020 caused the corn varieties to be more stressed. The performance assessment of maize varieties showed that the ADA 17.4, SYM-307, and Check-2 cultivars were more tolerant to stress, while the SYM-306 and SYM-305 varieties were more sensitive to ‘HS,’ and all other corn varieties were moderately sensitive to ‘HS.’ The ADA 17.4 and SYM-307 corn cultivars outperformed all other control and test corn cultivars. In addition to their physiological and biochemical tolerances, these cultivars are genetically tolerant to ‘HS.’ These results revealed that physiological parameters can be used to measure the stress and tolerance levels of plants during the tasseling period in the early stages of breeding. These physiological parameters could be used for a rapid selection of corn varieties in long-term breeding programs carried out using hundreds or even thousands of corn varieties. The CMD and LWP are easy and inexpensive traits to measure, providing a fast and effective selection opportunity at the beginning of breeding programs. This allows corn varieties to be classified as high tolerance, moderate tolerance, and sensitive corn varieties, allowing breeding programs to be created based on these classifications. Future studies should be carried out using a higher number of maize varieties, and more detailed physiological and biochemical analyses should be carried out to better understand the tolerance mechanisms of maize varieties under ‘HS’ conditions.

## Supplemental Information

10.7717/peerj.14141/supp-1 Supplemental Information 1 Raw data for 2020, 2021 and combined years. Years and combined years were subjected to variance analysis at 6 parameters investigated in the study, furthermore, statistical groups were formed according to the LSD test. Correlation test was performed between all traits determined in the study.

## Additional Information and Declarations

### Data Availability

The following information was supplied regarding data availability: The raw data is available in the Supplemental Files.
